# Technical considerations for using intravenous gadolinium-based-contrast-agent (GBCA) based MRI approaches to study cerebrospinal fluid (CSF) circulation and clearance

**DOI:** 10.1016/j.neuroimage.2025.121239

**Published:** 2025-04-24

**Authors:** Jun Hua, Yuanqi Sun, Yinghao Li, Xinyi Zhou, Yuhan Bian, Adrian Paez, Briana Meyer, Swati Rane Levendovszky

**Affiliations:** a Neurosection, Division of MR Research, Department of Radiology, Johns Hopkins University School of Medicine, Baltimore, MD, USA; b F.M. Kirby Research Center for Functional Brain Imaging, Kennedy Krieger Institute, Baltimore, MD, USA; c Department of Biomedical Engineering, Johns Hopkins University School of Medicine, Baltimore, MD, USA; d Department of Radiology, University of Washington, Seattle, WA, USA; e Department of Radiology, University of Kansas Medical Center, Kansas City, KS, USA

**Keywords:** Partial volume, Concentration, Extracranial, Intracranial, Cerebrovasculature, Reference signal, Intrathecal

## Abstract

Intravenously (IV) administered Gadolinium-based-contrast-agents (GBCAs) can enter the intracranial cerebrospinal-fluid (CSF) space via weak barriers between blood and CSF at multiple locations in the brain. This enables IV-GBCAs to be used as a tracer to study CSF circulation and clearance in the brain. With proper optimization, IV-GBCA induced signal changes can be robustly detected in various brain regions associated with CSF circulation. Nevertheless, whether these signal changes can be attributed to GBCA concentration changes in the CSF space should be interpreted with caution. This review attempts to discuss several technical challenges for using IV-GBCA MRI to study CSF circulation in the brain. First, it is critical to minimize the partial volume effects from the blood compartment as IV-GBCAs can present in both the blood and CSF compartments for a long time. Second, MRI approaches that can provide a quantitative measure of GBCA concentration in the CSF are preferred as raw MR signal intensities can often have a complicated relationship with GBCA concentration. Third, regions with intracranial and extracranial blood supply should be analyzed separately because GBCA distribution in regions with extracranial blood supply may not be a proper indicator for CSF clearance from the brain. Fourth, differences in the cerebrovasculature should be considered when comparing IV-GBCA concentration changes in the CSF in brain diseases. Finally, a proper reference signal needs to be established to calibrate longitudinal post-GBCA signals across sessions. Some of these issues may also apply to intrathecal GBCA MRI studies.

## 1. Introduction

Magnetic resonance imaging (MRI) approaches based on intravenously (IV) administered Gadolinium-based contrast agents (GBCAs) have been increasingly adopted to study cerebrospinal fluid (CSF) circulation and clearance in the human brain (Absinta et al., 2017; Bouzerar et al., 2013; Cao et al., 2020, 2023; Deike-Hofmann et al., 2019; Eide et al., 2020; Hsu and Chen, 2005; Kao et al., 2003; Kim et al., 2020; Lev and Schaefer, 1999; Naganawa et al., 2021a, 2020a, 2021b; Naganawa et al., 2020b, 2018; Naganawa and Taoka, 2022; Ohashi et al., 2021; Richmond et al., 2023; Smyth et al., 2024; Sudarshana et al., 2019; Sun et al., 2024; Taoka and Naganawa, 2020a, b, 2021; Wahlin et al., 2022; Wu et al., 2007). The enhanced sensitivity from GBCAs made it a useful method especially for regions with small CSF space. IV GBCA based MRI is currently the only FDA approved approach for contrast-enhanced MRI in human subjects, which has been routinely performed in the clinics for several decades. According to the American College of Radiology (ACR), Group II GBCAs, a newer generation of GBCAs compared to the linear GBCAs originally approved by the FDA in the 1980s, have an excellent safety profile and can even be used in patients with kidney disease (ACR, 2023). It is therefore safe and convenient to perform IV GBCA based MRI in various patient populations and even healthy human subjects.

Historically, IV GBCA based MRI has been primarily used to measure blood perfusion in the brain (Quarles et al., 2019), known as dynamic susceptibility contrast (DSC) MRI and dynamic contrast enhancement (DCE) MRI. IV GBCAs are generally not expected to cross an intact blood brain barrier (BBB). However, accumulating evidence has shown that IV GBCAs can get into the intracranial CSF space via loose barriers between blood and CSF at multiple locations in the brain (Antony et al., 2015; Berger et al., 2018; Deike-Hofmann et al., 2019; George et al., 2011; Jost et al., 2017; McKnight et al., 2020; Nehra et al., 2018; Smirniotopoulos et al., 2007; Sze et al., 1989; van Osch et al., 2024), which makes it possible to employ IV GBCAs as a tracer to study CSF circulation as well. For a comprehensive review of potential pathways for IV GBCAs to enter CSF circulation in the human brain, the readers are referred to (van Osch et al., 2024).

In this review, we attempt to discuss several technical challenges for using IV GBCA MRI to study CSF circulation in the brain. As many existing MRI pulse sequences for GBCA enhanced MRI were optimized for detecting IV GBCA induced signal changes in the blood, imaging parameters may need to be adjusted for CSF studies. MR signal changes can often be observed after IV GBCA administration in many regions associated with CSF circulation throughout the brain. However, whether these signal changes can be attributed to GBCA concentration changes in the CSF space should be interpreted with caution. Some of these issues may also be relevant to GBCA enhanced MRI studies using other delivery approaches such as intrathecal injection.

## 2. Partial volume effects from the blood compartment

**Fig. 1.**
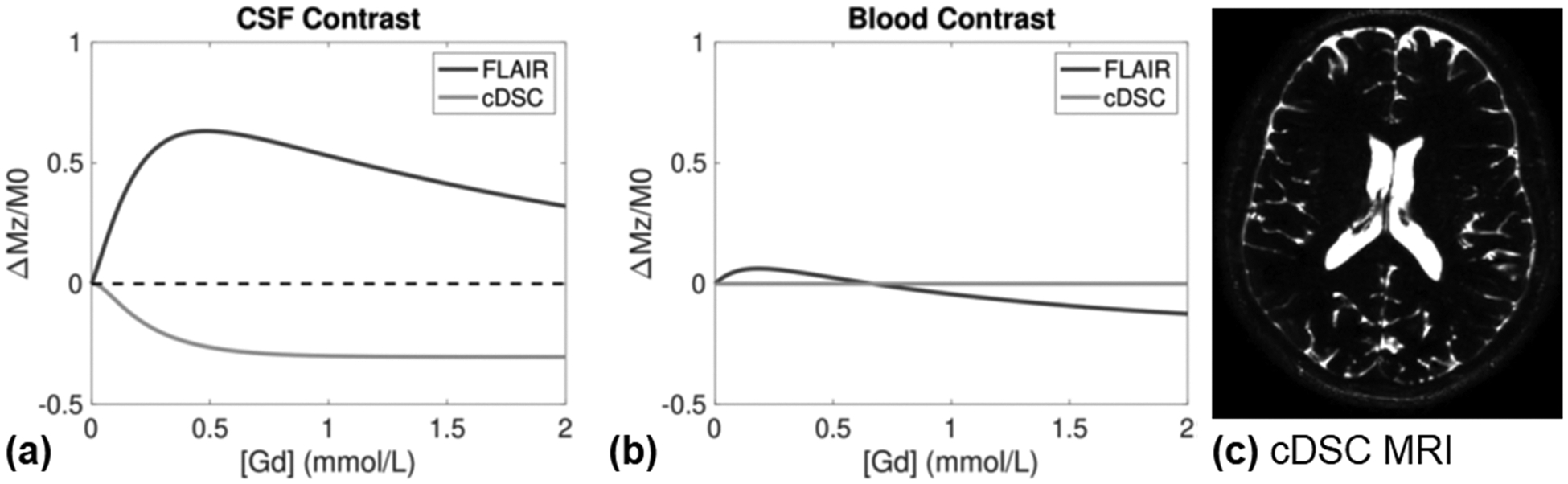
(a) Simulations of the relationship between GBCA concentration ([Gd]) and MR signal contrast (relative signal change between post- and pre-GBCA periods) in the CSF in FLAIR and cDSC MRI. (b) Simulations of the relationship between GBCA concentration and MR signal contrast (relative signal change between post- and pre-GBCA periods) in the CSF in FLAIR and cDSC MRI. Imaging parameters used in the simulation: **FLAIR**: repetition time (TR)/inversion time (TI)/ TE = 6000/2000/180 ms, three dimensional (3D) turbo spin echo (TSE) readout, TSE factor = 70; **cDSC**: TR/TE/echo spacing (ES) = 10,000/1347/3.2 ms, 3D TSE readout, TSE factor = 1024, single shot. For additional details, please refer to Sun et al. (2024). **(c)** A representative cDSC MRI image in a healthy human brain.

In IV GBCA based MRI approaches, GBCAs can present in both the blood and CSF compartments. The first pass of GBCAs in the blood during the bolus phase following IV injection usually takes approximately 20–30 s, but low concentration of GBCAs in the range of 0–0.5 mmol/L, which is similar to the GBCA concentration in CSF after IV injection (Ringstad et al., 2023), can remain in the blood circulation from 25–30 min up to several days (Berger et al., 2018; Nehra et al., 2018; Uh et al., 2009). For instance, a recent study showed substantial GBCA enhanced MR signals in the sagittal sinus at 6 h after IV injection (Richmond et al., 2023). Even with sub-millimeter spatial resolution, a voxel can still have substantial partial volume effects from blood and CSF. This means that GBCA induced signal changes measured from the voxel can originate from either the blood, CSF, or both compartments. Many sequences widely used in the DSC and DCE MRI literature such as T1-weighted fast gradient-echo (GRE) sequences (Ahn et al., 2019; Eide and Ringstad, 2019; Eide et al., 2018; Gaberel et al., 2014; Goulay et al., 2017; Iliff et al., 2013; Lee et al., 2018, 2015; Ma et al., 2019; Ringstad and Eide, 2020; Ringstad et al., 2018, 2017; Taoka and Naganawa, 2020a), T1-weighted fast spin-echo (FSE) sequences (Da Mesquita et al., 2018; Eide et al., 2018; Magdoom et al., 2019; Ringstad and Eide, 2020), and inversion recovery based sequences including fluid-attenuation inversion recovery (FLAIR) (Absinta et al., 2017, 2015; Ahn et al., 2019; Albayram et al., 2022; Lee et al., 2018; Naganawa et al., 2020a; Ringstad and Eide, 2020) and black blood (Absinta et al., 2017) MRI can have substantial signal contributions from both the blood and CSF compartments. For instance, FLAIR MRI has been used in several recent studies to measure IV GBCA distribution in the human brain (Absinta et al., 2017; Naganawa et al., 2018). As shown in Fig. 1a&b, when GBCAs present in the blood or the CSF compartment, they can induce FLAIR signal changes in the respective compartment. While the post-GBCA FLAIR signal in the CSF increases compare to the pre-GBCA FLAIR signal, the post-GBCA FLAIR signal in the blood can sometime decrease depending on the concentration of GBCA in blood.

To minimize partial volume effects from blood, T2-weighted FSE sequences with a long echo time (TE) can be used (Cao et al., 2020; Magdoom et al., 2019) (please see the next section for a discussion on T1-weighted and T2-weighted sequences). Since CSF has a substantially longer T2 relaxation time compared to the other components of the brain (parenchyma/blood T2~100 ms; CSF T2~1000 ms) (Cao et al., 2020), MR images acquired at a sufficiently long TE should have predominant signal contribution from the CSF, with minimal signals from the other components due to much faster T2 decay. One example is the recently developed dynamic susceptibility contrast in the CSF (cDSC) MRI approach (Cao et al., 2020). A TE of >1000 ms is applied in cDSC MRI (Cao et al., 2020). Fig. 1a&b show that with a proper optimization of the imaging parameters, GBCA-induced MR signal changes in cDSC MRI are minimal in blood, but are still sizable in the CSF. The resulting cDSC images can provide a map of GBCA distribution in the CSF alone, with signals in each voxel reflecting the amount of CSF occupying the voxel (Fig. 1c). More recently, the cDSC MRI method was expanded using a dual-echo sequence, termed “dynamic dual-spin-echo perfusion (DDSEP) MRI”, so that perfusion parameters related to both the blood and lymphatic vessels can be measured in one single scan in human subjects (Cao et al., 2023). One caveat when using long TE T2-weighted sequences is that it is susceptible to artifacts caused by fast or pulsatile CSF flow in regions such as the aqueduct. Therefore, the use of long TE T2-weighted sequences in IV GBCA based MRI for CSF studies should be restricted to areas with relatively slow CSF distribution. The bulk CSF flow at the aqueduct is commonly measured using phase-contrast (PC) MRI.

**Fig. 2.**
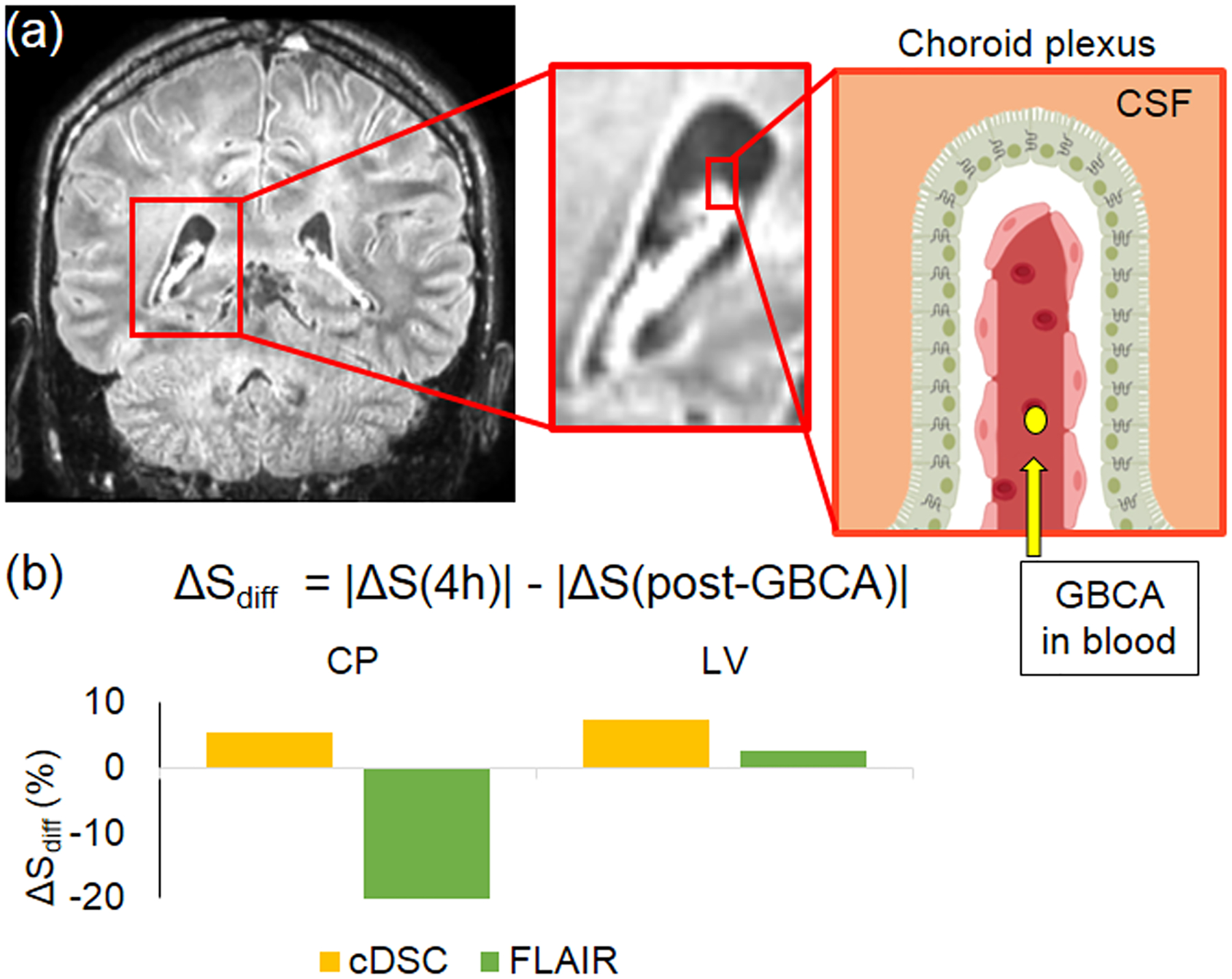
Partial volume effects from blood and CSF in FLAIR MRI. **(a)** A FLAIR MRI image showing the choroid plexus. The choroid plexus is highly vascularized. IV GBCAs can cross the blood-CSF barrier in the choroid plexus to enter the ventricular CSF. Reproduced with permission. **(b)** Differences of GBCA-induced signal changes (ΔS) from the CSF between 4 h and immediate post-GBCA periods measured by cDSC and FLAIR MRI, respectively (*n* = 10). CP: choroid plexus; LV: lateral ventricle. For additional details, please refer to Sun et al. (2024).

The partial volume effects from blood can be particularly problematic for the interpretation of longitudinal IV GBCA results in which GBCA concentration in both blood and CSF can vary over time. Here we provide one example from a recent longitudinal study. Sun et al. (2024) performed both FLAIR and cDSC MRI in healthy human subjects to measure IV GBCA distribution in the choroid plexus and lateral ventricle at two time points after IV injection: immediate and 4 h post-GBCA. The choroid plexus is highly vascularized which leads to substantial partial volume effects from blood, whereas voxels in the rest of the lateral ventricle contain predominantly CSF (Fig. 2a). In the lateral ventricle excluding choroid plexus, the difference of magnitudes of GBCA-induced FLAIR signal changes between the 4 h and immediate post-GBCA time points showed a similar trend as the magnitude of cDSC signal changes (Fig. 2b). In contrast, in the choroid plexus, the difference of magnitudes of GBCA-induced signal changes between the 4 h and immediate post-GBCA time points showed an opposite trend between cDSC and FLAIR MRI. Such partial volume effects from blood in FLAIR can result in misleading interpretation of GBCA concentration changes in the CSF over time. For instance, if one attributes the reduced FLAIR signal changes at 4 h in the choroid plexus entirely to the GBCAs in CSF, the conclusion would be GBCA concentration in CSF decreased at 4 h compared to immediate post-GBCA. However, what actually happened may be that GBCA concentration in blood decreased significantly (close to zero) at 4 h but GBCA concentration in CSF increased at 4 h compared to immediate post-GBCA, leading to an overall reduced FLAIR signal change. This example demonstrates that in longitudinal studies, as the concentrations of GBCA in blood and CSF change over time, the overall longitudinal signal changes detected in the voxel may have a different trend from the GBCA concentration changes in each compartment. Therefore, an MRI method that can provide a clean CSF signal without partial volume effects from blood such as cDSC MRI is strongly preferred for IV GBCA based MRI studies of CSF circulation.

## 3. The relationship between GBCA concentration and MR signal intensities may not be straightforward

**Fig. 3.**
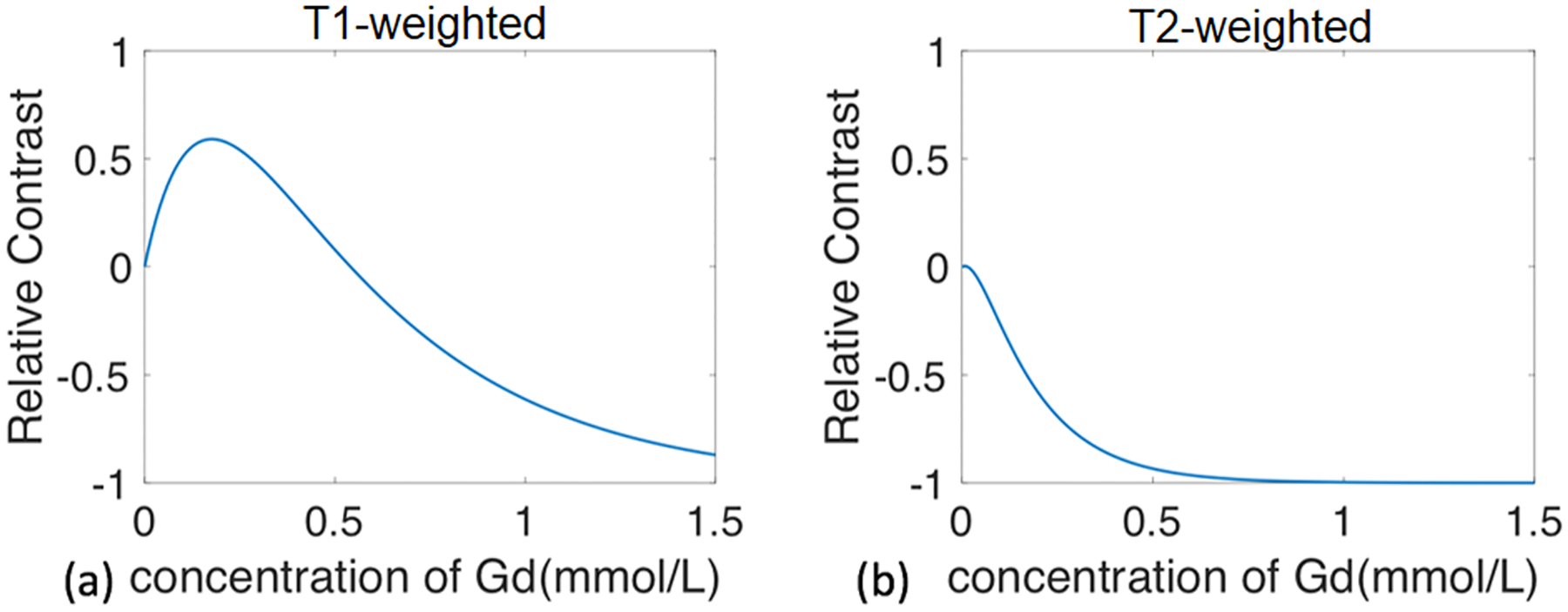
Simulation of the relationship between GBCA concentration and MR signal contrast (relative signal change between post- and pre-GBCA periods). **(a)** GBCA-induced signal changes in a T1-weighted FSE sequence (3D TSE, TR/TE=2500/482 ms) are displayed as a function GBCA concentration. **(b)** GBCA-induced signal changes in a T2-weighted FSE sequence (3D TSE, TR/TE=9000/1391 ms) are displayed as a function GBCA concentration. For additional details, please refer to Cao et al. (2020).

The presence of GBCAs alters both T1 and T2 values of the medium (blood or CSF), the reciprocals of which (R1=1/T1 and R2=1/T2) are proportional to the concentration of GBCA. The observed post-GBCA MR signal change, however, does not have such a straightforward relationship with GBCA concentration as it reflects the combined effects from both T1- and T2-shortening induced by GBCAs. Depending on the pulse sequences and imaging parameters applied, hyper-intensities or greater post-GBCA MR signals do not always correspond to higher GBCA concentration. This is well known for example in kidney and bladder MRI especially when using T1-weighed sequences (Elster et al., 1990). Fig. 3 showed an example for the relationship between GBCA concentration and the post-GBCA MR signal intensity change simulated for T1-weighted and T2-weighted FSE sequences. In the T1-weighed FSE sequence, post-GBCA signal intensity change had a biphasic relationship with GBCA concentration. When GBCA concentration is above a certain level, its T2-shortening effect starts to dominate the T1-shortening effect, resulting in an overall decrease of the T1-weighed signal. In the T2-weighted FSE sequence, the imaging parameters were chosen so that the post-GBCA signal intensity change had a monotonic relationship with GBCA concentration (Cao et al., 2020). Therefore, when choosing MRI pulse sequences and imaging parameters for IV GBCA studies, it is critical to understand the relationship between the expected GBCA-induced signal changes and GBCA concentration. Note that such relationship can also vary with the range of expected GBCA concentration in the CSF and blood, which can be very different for various GBCA administration procedures such as intravenous and intrathecal. To avoid such uncertainty, instead of using MR signal intensity changes to infer GBCA concentration change, methods that can quantify GBCA concentration in the CSF are strongly preferred for CSF studies using IV GBCA based MRI.

**Fig. 4.**
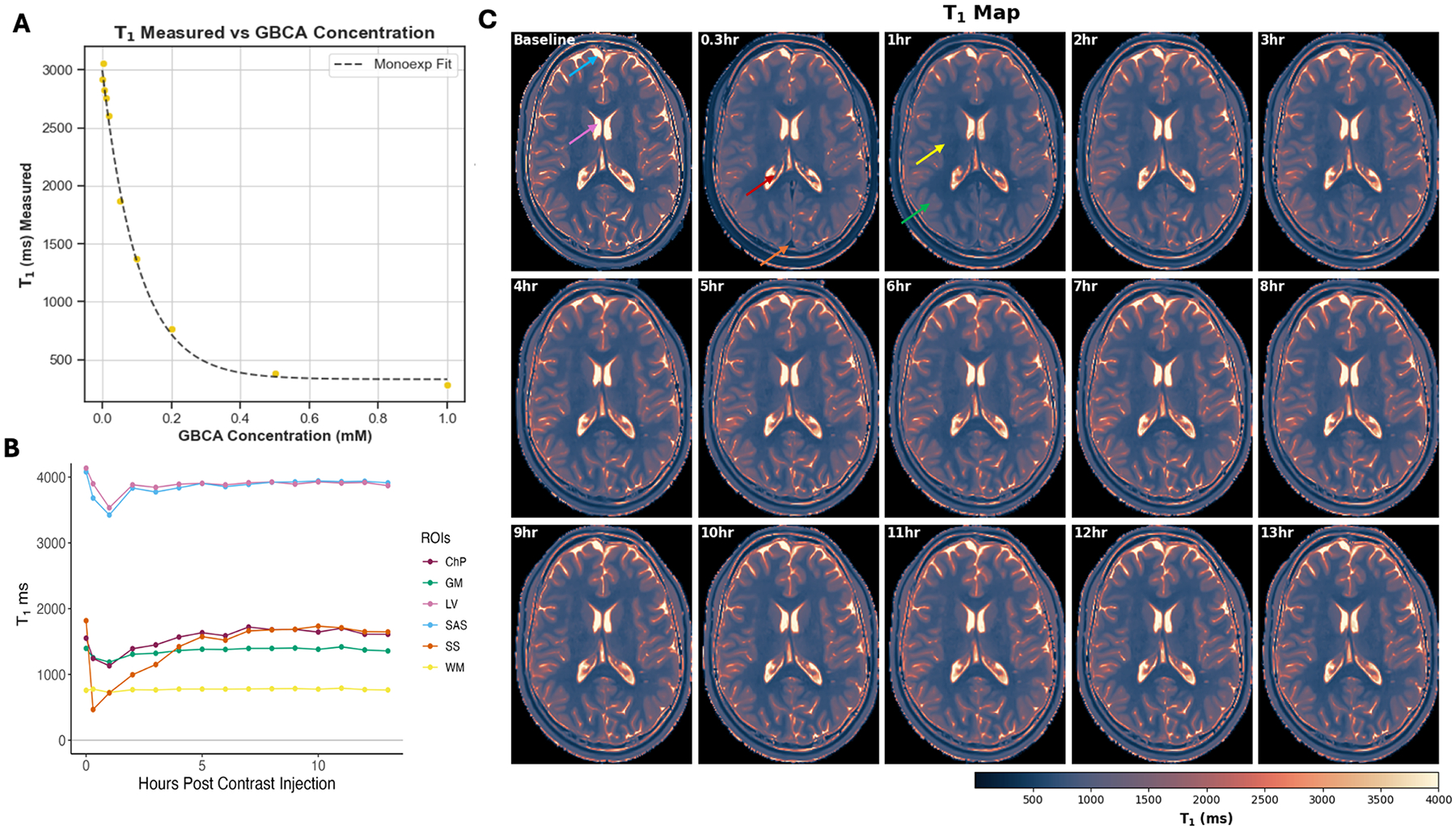
T1 mapping to quantify GBCA concentration in blood and CSF. **(a)** Validation of the method in phantoms with various GBCA concentration. T1 values show a mono-exponential decay with GBCA concentration. (**b,c**) T1 values and T1 maps (a single slice) acquired in a 25-year-old female participant before and after IV GBCA administration. T1 values in the sagittal sinus (SS) remained shortened at ~20 min post-GBCA. In the CSF compartments (lateral ventricle or LV, and subarachnoid space or SAS), maximum T1 shortening occurred at ~1 h post-GBCA, which is slightly delayed compared to the SS. T1 shortening in the blood and CSF compartments can still be observed at 5 h post-GBCA.

T1 mapping is commonly used in the DSC and DCE MRI literature to quantify GBCA concentration in the blood (Quarles et al., 2019). It has also been used in IT (Ringstad et al., 2023) and IV (Meyer et al., 2025; Wahlin et al., 2022) GBCA based MRI to quantify GBCA concentration in the CSF. As the T2-weighing is removed in T1 mapping, the T1 values no longer have the biphasic relationship with GBCA concentration like the T1-weighted signal. Fig. 4 shows a recent study (Meyer et al., 2025) using the 3D Quantification Using an Interleaved Look-Locker Acquisition Sequence with a T2 preparation pulse (3D QALAS) approach to quantify voxel-wise T1 values in various human brain regions at multiple time points after IV GBCA administration. GBCA concentration was estimated from the T1 values. In a healthy subject, GBCA concentration was approximately 0.017 mmol/L in the blood (sagittal sinus) and 0.002 mmol/L in the subarachnoid space (SAS) CSF at 5 h post-GBCA.

The choice of method also depends on the required spatial and temporal resolution. Sub-millimeter spatial resolution is usually desired for imaging CSF distribution and clearance in small structures such as the SAS and dura mater. The T2-weighted cDSC MRI method can be used to track dynamic changes in GBCA concentration in the CSF with a temporal resolution of < 10 s, a sub-millimeter spatial resolution and a whole-brain coverage (Cao et al., 2020). Typical T1- and T2-mapping approaches with a sub-millimeter spatial resolution usually take approximately 5–10 min to acquire one whole-brain volume (Meyer et al., 2025; Wahlin et al., 2022).

## 4. Intracranial and extracranial blood supply

**Fig. 5.**
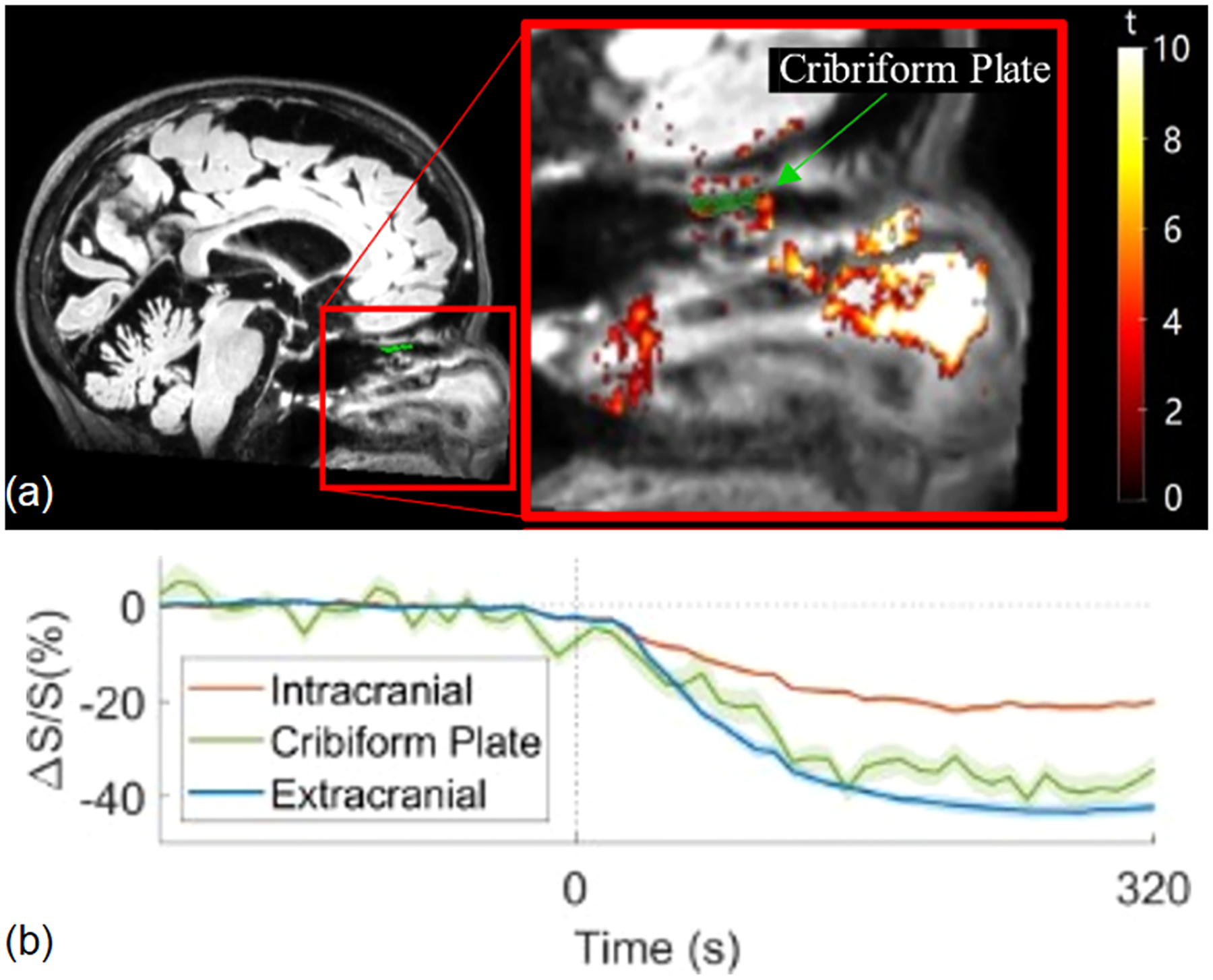
**(a)** Maps of voxels showing significant IV GBCA induced MR signal changes in the olfactory regions. The t-scores of significant voxels are superimposed on the post-GBCA images in the olfactory regions. The regions superior and inferior to the cribriform plate (green) are considered intra- and extra-cranial olfactory regions, respectively. **(b)** Average time courses of GBCA-induced signal changes (ΔS/S) from all intracranial and extracranial ROIs and the cribriform plate. GBCAs were administered at 0 s. The shade indicates the inter-ROI standard errors. For additional details, please refer to Zhou et al. (2024).

As IV GBCAs are directly injected into the bloodstream, it is important to consider the blood supply to the target region when interpreting IV GBCA distribution and clearance data. Unlike intracranial blood vessels, extracranial blood vessels do not have a BBB. Therefore, IV GBCAs may enter and clear from regions with extracranial blood supply directly from the peripheral route without passing through the brain. Thus, IV GBCA distribution and clearance in regions with extracranial blood supply may not be a proper indicator for CSF circulation and clearance in the brain. For instance, the olfactory pathway is considered an important route for CSF clearance from the brain. Regions along the olfactory pathway are separated by the cribriform plate. Olfactory regions superior to the cribriform plate such as the olfactory bulb are intracranial olfactory regions supplied by branches of the internal carotid artery (ICA) (Hendrix et al., 2014). On the other hand, olfactory regions inferior to the cribriform plate such as the nasal mucosa are extracranial olfactory regions supplied by branches arising from the external carotid artery (ECA) (Hendrix et al., 2014). Zhou et al. demonstrated that after IV GBCA administration, extracranial olfactory regions showed spatially more extensive MR signal changes with greater magnitude than intracranial olfactory regions (Fig. 5) (Zhou et al., 2024). Nevertheless, the GBCA distribution and clearance in the extracranial olfactory regions may occur primarily via the peripheral routes. Therefore, caution should be taken when relating IV GBCA induced MR signal changes in extracranial regions to CSF circulation and clearance in the brain (de Leon et al., 2017; Sennfalt et al., 2023). This also stresses the importance of separating intra- and extra-cranial areas in the analysis in CSF studies using IV GBCA based MRI approaches.

## 5. Influence from changes in the cerebrovasculature

Similar to the arterial input function in DCE MRI, the amount of IV GBCAs delivered into the CSF is determined by the status of the cerebrovasculature. Therefore, changes in the cerebrovasculature such as cerebral blood flow (CBF), blood barrier permeability, and cerebrovascular reactivity (CVR) can have substantial influence on the distribution and clearance of IV GBCAs in the CSF. A number of studies have demonstrated impaired CSF clearance in various brain diseases using IV GBCA based MRI (van Osch et al., 2024). On the other hand, it is also well-known that the cerebrovasculature is affected in many of these diseases. Few studies to date have examined the relationship between changes in parameters related to the cerebrovasculature and the observed changes in IV GBCA distribution and clearance in the CSF in brain diseases. Whether the decreased clearance of IV GBCAs from the CSF can be at least partially explained by the abnormal CBF, permeability and CVR remains to be investigated. Thus, it is highly desirable to include some measures of the homeostasis of cerebrovasculature in disease studies using IV GBCAs, and proper models and analysis approaches should be developed and applied.

## 6. Reference signal to calibrate post-GBCA MR signals across longitudinal sessions

The signal intensities in MR images acquired using the same sequence from the same subject can vary substantially across longitudinal sessions. Many factors such as receiver gain, B1 homogeneity, head position in the coil, coil loading and matching, preparation and calibration steps including power optimization, shimming and reference scans can contribute to this variation. Some of these effects can be corrected for using vendor-provided scaling factors, but such correction is usually not sufficient to account for all the variables. One commonly applied approach is to normalize the longitudinal signal intensities with signals from an internal reference region in the brain. Such reference region should ideally be relatively large and homogeneous, and have a similar B0 and B1 field fluctuation as most areas in the brain. However, in IV GBCA MRI studies, almost all brain regions including deep gray and white matter and the eye region will eventually show some degree of GBCA-induced signal changes (van Osch et al., 2024), making it very difficult to find a proper reference region within the brain. Smaller structures such as intracranial fat may have very different B0 and B1 fields compared to the rest of the brain. External objects such as fiducial markers or water bags can be placed around the participant’s head to provide a reference region. Nevertheless, as the B0 and B1 fields can alter significantly in areas outside the brain, the signals from these external objects may not be a proper reference for regions in the brain. Even when quantitative methods such as T1 or T2 mapping are adopted, B1 inhomogeneity is a major error source for T1 and T2 quantification, which can fluctuate substantially over time. Therefore, it is critical to evaluate the longitudinal stability of the aforementioned approaches, so that the fluctuations of the reference signal do not overwhelm small GBCA-induced MR signal changes over time. Note that this issue is not limited to IV GBCA MRI studies but also applies to GBCA-enhanced MRI studies using other delivery routes such as intrathecal injection. One promising technique is the ERETIC (Electric REference To access In vivo Concentrations) method (Akoka et al., 1999; Franconi et al., 2002; Thapa et al., 2022; Ziarelli et al., 2008; Zoelch et al., 2017), which injects an electronic reference signal into the receive coil during image acquisition to be used as the internal calibration standard. But the ERETIC approach requires hardware modification, and its longitudinal reliability needs to be assessed for IV GBCA MRI studies.

## 7. Conclusions

IV GBCAs can be used as a tracer to study CSF circulation and clearance in the brain. The concentration of GBCAs in the CSF when administered intravenously is lower than intrathecal GBCAs (Ringstad et al., 2023). But with proper optimization of imaging parameters, IV GBCA induced MR signal changes can be robustly detected in various regions of the brain associated with CSF circulation. Meanwhile, several technical issues should be considered when interpreting such signal changes as multiple factors other than GBCA concentration changes in the CSF space can contribute to them. First and foremost, it is critical to use MRI methods that minimizes the partial volume effects from the blood compartment which can cause misleading results especially for longitudinal studies. It is preferred to adopt MRI approaches that can provide a quantitative measure of GBCA concentration in the CSF as raw MR signal intensities can often have a confounding relationship with GBCA concentration. Regions with intracranial and extracranial blood supply should be analyzed separately because IV GBCAs from extracranial blood supply may not pass through the brain. Differences in the cerebrovasculature should be considered when comparing IV GBCA concentration changes in the CSF among different physiological and pathological conditions. Finally, a proper reference signal with excellent longitudinal stability needs to be established to calibrate post-GBCA MR signals across multiple longitudinal time points.

## Data Availability

Data supporting the findings of this review article are all from previously published studies and are available in the cited references. No new data were generated or analyzed for this review.
